# Fungemia due to *Lachancea fermentati*: a case report

**DOI:** 10.1186/1471-2334-14-250

**Published:** 2014-05-10

**Authors:** Anne-Marie Leuck, Meghan K Rothenberger, Jaime S Green

**Affiliations:** 1 Department of Medicine, University of Minnesota, D-416 Mayo Building, MMC 250, 420 Delaware St. SE, Minneapolis, MN 55455, USA

**Keywords:** *Lachancea fermentati*, Fungemia, Opportunistic pathogen

## Abstract

**Background:**

*Lachancea fermentati* is an environmental yeast that is also used in the fermentation of alcoholic drinks. It has not previously been described as a human pathogen although the closely related yeast, *Saccharomyces boulardii*, can cause fungemia. Here we report a case of *L. fermentati* acting as a pathogen in a septic patient with cultures positive from blood, peritoneal fluid, bile, and sputum.

**Case presentation:**

A 36 year-old Caucasian man was hospitalized with acute alcoholic hepatitis complicated by *Escherichia coli* spontaneous bacterial peritonitis. Three days after admission, he developed new fevers with sepsis requiring mechanical ventilation and vasopressor support. He was found to have a bowel perforation. Cultures from blood, peritoneal fluid, and sputum grew a difficult-to-identify yeast. Micafungin was started empirically. On hospital day 43 the yeast was identified as *L. fermentati* with low minimum inhibitory concentrations (by Epsilometer test) to all antifungals tested. Micafungin was changed to fluconazole to complete a 3-month course of therapy. Serial peritoneal fluid cultures remained positive for 31 days. One year after his initial hospitalization the patient had ongoing cirrhosis but had recovered from fungemia.

**Conclusion:**

This case demonstrates the need for clinicians to consider host factors when interpreting culture results with normally non-pathogenic organisms. In this immunocompromised host *L. fermentati* caused disseminated disease. We believe his hobby of brewing alcohol led to colonization with *L. fermentati*, which then resulted in invasive disease when the opportunity arose.

## Background

Non-*Candida* fungemia has become increasingly problematic among immunocompromised hosts, and fungal genera such as *Trichosporon*, *Cryptococcus*, *Rhodotorula, Malassezia*, and *Blastoschizomyces* have become recognized as opportunistic pathogens [1-3]. The genus *Saccharomyces* has also been described as a human pathogen [4-6]. We describe a case of fungemia and sepsis due to the yeast *Lachancea fermentati*, a species closely related to the saccharomycetes. The known potential of *Saccharomyces* to act as a pathogen suggests that the related species, *L. fermentati,* may also have the ability to cause disease in the appropriate clinical setting.

## Case presentation

**Figure 1:**
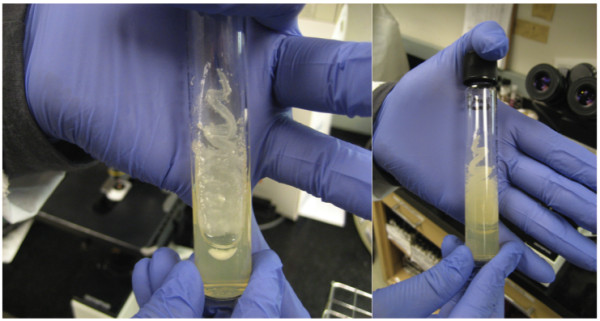
**Front and back view of *L. fermentati *** **grown on Sabouroud dextrose agar showing the creamy white colonies typical of yeast.**

A 36 year-old man with acute alcoholic hepatitis was hospitalized due to *Escherichia coli* peritonitis attributed to spontaneous bacterial peritonitis. His social history was notable for brewing his own alcohol and alcohol abuse. He initially improved symptomatically, but three days after admission he developed a fever to 38.1°C and multi-organ failure requiring mechanical ventilation, continuous renal replacement therapy, and vasopressor support. He clinically stabilized with intensive critical care support and initiation of empiric antibiotics (vancomycin, meropenem, and micafungin). Blood cultures at the time of admission were negative. One of four blood culture bottles drawn at the time of clinical decompensation became positive for yeast after 12 hours of incubation (BacT Alert System®, Biomerieux, Durham, NC). Sputum and peritoneal fluid cultures collected the following day also grew the same yeast. Culture showed creamy white colonies on Sabouroud-dextrose agar (Figure 1). The yeast was not immediately identifiable by the clinical microbiology lab.

During the two-month admission, he was found to have loculated ascites associated with a fistulous connection to bowel. Multiple abdominal abscess and peritoneal fluid cultures had polymicrobial growth (Table 1). Despite the intermittent presence of other organisms, every peritoneal culture over a one-month period, nine in total, grew the same difficult-to-identify yeast. This same yeast also grew from three sputum cultures (Table 1). The yeast isolated from sputum was felt to more likely represent colonization than infection. The patient had pleural effusions attributed to his ascites but he did not have a clinical pneumonia. Urine cultures remained negative. Medical management included aggressive drainage of the abdominal fluid collections and continuation of broad-spectrum antimicrobials including meropenem, vancomcyin, and micafungin. Over time, the patient slowly improved with resolution of the abdominal pain, fever, leukocytosis, and abdominal abscesses.

**Table 1:** Summary of positive cultures during hospitalization

**Hospital day of culture**^**a**^	**Site of isolation**	**Organism(s) isolated**
Day 3	Peritoneal fluid	*Eggerthalla lenti*
Day 5	Blood	*Lachancea fermentati*
Day 6	Sputum	*L. fermentati*
	Peritoneal fluid	*L. fermentati, Enterococcus* spp., *Lactobacillus*
Day 8	Sputum	*L. fermentati, Candida rugosa*
Day 9	Peritoneal fluid	*L. fermentati*
Day 10	Peritoneal fluid	*L. fermentati*
Day 11	Peritoneal fluid	*L. fermentati*
Day 12	Peritoneal fluid	*L. fermentati*
Day 15	Peritoneal fluid	*L. fermentati*
	Sputum	*L. fermentati*
Day 16	Bile	*L. fermentati,* coagulase negative staphylococcus
Day 24	Peritoneal fluid	*L. fermentati*
Day 36	Abdominal abscess	*L. fermentati, Candida parapsilosis, Bacteroides* spp*., Pseudomonas aeruginosa, Clostridium* spp.
Day 46	Sputum	*C. parapsilosis,* coagulase negative staphylococcus
Day 50	Sputum	*C. parapsilosis,* coagulase negative staphylococcus

^a^Note that the patient also had a peritoneal fluid culture positive for *Escherichia coli* prior to admission.

On hospital day 43, the yeast isolated from blood, peritoneal fluid, bile, and sputum was identified as *Lachancea fermentati* by the Fungus Testing Laboratory at the University of Texas Health Science Center. Identification was based on cornmeal agar morphology, cycloheximide susceptibility studies, temperature studies, and sequencing of the D1/D2 regions of 28 s rDNA. No susceptibility breakpoints are available for *L. fermentati*, but the minimum inhibitory concentration (MIC) was low for all antifungal agents tested (Table 2). When MIC values were known, micafungin was changed to fluconazole 100 mg daily (approximately 3 mg/kg, the renal-adjusted dose equivalent of 6 mg/kg). He completed 6 weeks of therapy with micafungin and an additional 6 weeks of fluconazole, for a total 3 months of antifungal therapy. One year after his initial presentation he remained abstinent from alcohol and fully recovered from the fungemia. He does have cirrhosis and at the time of his last clinic visit he continued on ciprofloxacin for spontaneous bacterial peritonitis prophylaxis.

**Table 2:** ***Lachancea fermentati*** **minimum inhibitory concentrations (MIC) by Epsilometer test (E-test)**

**Antifungal agent**	**MIC by E-test (ug/mL)**
Amphotericin B	0.064
Caspofungin	0.38
Fluconazole	0.75
Itraconazole	0.064
Voriconazole	0.016

## Conclusions

*L. fermentati* is an environmental, saprophytic yeast found in decaying material. In its natural setting, *Lachancea* colonizes leaf surfaces and may provide a natural buffer against plant pathogens [7]. *Lachancea* species, in combination with *Saccharomyces cerevisiae*, are commonly used in the fermentation process to make wine and cachaca (a drink made from fermented sugar) [8,9], and have been shown to enhance the quality and aroma of these beverages [10].

While *L. fermentati* has been recognized as a component of fermented drinks, it may be more pervasive than previously thought. *L. fermentati* was found in more than 50% of olive oil mills tested [5], and an investigation of commercially available drinks in Brazil also found *L. fermentati* in coconut juice and reconstituted fruit juices [11]. A species with a close evolutionary relationship to *L. fermentati*, *L. thermotolerans*, is present on the leaves of deciduous trees in the autumn when fruit would be harvested [7]. With this increasing recognition of *Lachancea*’s environmental presence, clinicians and clinical microbiologists should be aware of its pathogenic potential.

Recent progress has also been made in the classification of *Lachancea* yeasts. *L. fermentati*, formerly known as *Zygosaccharomyces fermentati,* is related to the genus *Saccharomyces*. Prior to DNA sequencing, classification of these organisms was based on morphologic and phenotypic characteristics. The advent of nuclear-based rDNA sequencing technology, coupled with multigene-based phylogenetic analyses, has led to reclassification of the 11 clades of *Saccharomycetaceae.* The genus *Lachancea* consists of five species, *L. cidri, L. fermentati, L. kluyveri, L. thermotolerans* and *L. waltii.* The organism is further characterized by vegetative reproduction with multilateral budding on a narrow base and fermentation of glucose in addition to at least one other sugar [12]*.* Based on comparative analyses of rDNA sequences and molecular karyotyping of *Lachancea* species, the yeast appears to have eight chromosomes [13], and its preferred growing temperature is 25-37°C [14]. This range includes normal human body temperature and suggests that *L. fermentati* could act as a pathogen.

Of interest, one of this patient’s early paracenteses grew *Eggerthella lenta*, which is associated with the gastrointestinal flora of wine drinkers [15]. This patient did indeed brew his own alcohol. Taken together, the presence of two microorganisms associated with fermented drinks suggests that his microbiome may have been affected by his alcohol brewing and consumption, resulting in infection with an unusual pathogen, *L. fermentati.* In a similar case, *S. cerevisiae* (baker’s yeast) caused disseminated disease in a baker’s wife with leukemia, which was thought to be associated with occupation-related colonization [16].

To the best of our knowledge, this is the first case reporting *L. fermentati* as a human pathogen. This case of *L. fermentati* fungemia highlights the ability of non-pathogenic microorganisms to cause disease in unusual situations depending on host risk factors and clinical circumstances. If this patient had not been fungemic and septic, this yeast might have been considered a colonizing species. However, in this immunocompromised host with a perforated viscus, it acted as a pathogen and required treatment.

## Consent

Written informed consent was obtained from the patient for publication of this case report. A copy of the written consent is available for review by the Editor of this journal.

## Competing interests

The authors declare that they have no competing interests.

## Authors’ contributions

AML wrote the manuscript. JG and MR contributed patient data and were involved in editing the manuscript. All authors read and approved the final manuscript.

## Pre-publication history

The pre-publication history for this paper can be accessed here:

http://www.biomedcentral.com/1471-2334/14/250/prepub
